# Classic and rare manifestations of multiple osteoma: A case report

**DOI:** 10.1016/j.ijscr.2023.108713

**Published:** 2023-08-25

**Authors:** Yuni Artha Prabowo Putro, Rahadyan Magetsari, Kartika W. Taroeno-Hariadi, Ery Kus Dwianingsih, Amri Wicaksono Pribadi, Karisa Kartika Sukotjo

**Affiliations:** a Department of Orthopedics and Traumatology, RSUP Dr. Sardjito Hospital, Jl. Kesehatan Sendowo No.1, Sleman 55281, D.I.Yogyakarta, Indonesia; b Faculty of Medicine, Public Health and Nursing, Universitas Gadjah Mada, Jl. Farmako, Sendowo, Sekip Utara, Sleman 55281, D.I.Yogyakarta, Indonesia; c Department of Internal Medicine, Faculty of Medicine, Public Health and Nursing, Universitas Gadjah Mada/Dr Sardjito General Hospital, Jl. Farmako, Sendowo, Sekip Utara, Sleman 55281, D.I.Yogyakarta, Indonesia; d Department of Anatomical Pathology, Faculty of Medicine, Public Health and Nursing, Universitas Gadjah Mada/Dr Sardjito General Hospital, Jl. Farmako, Sendowo, Sekip Utara, Sleman 55281, D.I.Yogyakarta, Indonesia; e Department of Radiology, Faculty of Medicine, Public Health and Nursing, Universitas Gadjah Mada, Jl. Farmako, Sendowo, Sekip Utara, Sleman 55281, D.I.Yogyakarta, Indonesia

**Keywords:** Multiple osteoma, Oncology, Benign bone tumor, Case report

## Abstract

**Introduction and importance:**

Osteoma is a benign tumor that can arise from compact or cancellous bone and is more commonly found in the face or skull. The incidence of osteoma believed to be underreported as most are asymptomatic. To date, the best modality to diagnose osteoma is CT scan. We report a unique case of osteoma presenting with cranial and extracranial manifestations and highlight the importance of bone survey in evaluating patients with osteoma.

**Case presentation:**

A 26-year-old female complained of bilateral pain in the jawbone and several areas of her head. On physical examination, there were several masses in the head with the largest on the left mandible measuring 5.6 × 6.0 × 4.5 cm from MSCT examination. Hemi-mandibulectomy, histopathological and cytopathology examination were performed on the tissue obtained from the left mandible which concluded osteoma. Post-operative bone survey was performed and found osteoma on left ulna and bilateral fibula. Suspected Gardner syndrome with multiple osteoma manifestation was excluded from normal results of colon in-loop examination. We conservatively monitored the patient and most recent 6-month follow-up found no complaint nor changes in the extracranial osteoma manifestation on left ulna and both fibulas.

**Clinical discussion:**

The benign tumor osteoma is incredibly uncommon to present both intra and extracranially. We suggest thorough skeletal studies such as bone survey to be performed as they are crucial in the full evaluation of patients with multiple osteomas. Osteoma treatment is based on the patient's symptoms, surgery for patients with symptoms and periodic monitoring for asymptomatic patients.

**Conclusion:**

It is necessary to consider radiological modality for diagnosing osteoma patients. The majority of osteomas are asymptomatic and the choice of radiological examination sometimes still misses the lesion. It is important to evaluate histologically if the lesion difficult to diagnose.

## Highlights

• Osteoma is a benign tumor that rarely manifests cranial and extracranial.
• CT scanning is the superior radiological modality for the diagnosis of osteoma.
• Bone survey is important in patients with multiple osteomas.
• Multiple osteomas are often associated with gardener syndrome.
• Management of osteoma is based on its complaint, symptomatic or asymptomatic.

## 1 Introduction

Osteoma was first reported by Jaffe in 1935 [1]. Osteoma is a benign tumor characterized by proliferation of cancellous or compact bone and is more commonly found in the face or skull [[2], [3], [4]]. The predilection took place due to its formation from intramembranous ossifications. Currently, osteoma is divided into 3 types based on its origin, central osteoma, peripheral osteoma and extra skeletal soft tissue osteoma. Meanwhile, osteoma can histologically be divided into subgroups of compact (ivory), spongious (trabecular) and mixed types [2,5]. Clear margin of osteoma lesions rarely extends into the medullary cavity, although separate development of osteoma directly to this area may occur and known as bone island [2,4].

Osteomas are generally asymptomatic, although in some cases pain, facial asymmetry and joint disturbances can be found [5]. In patients with 3 lesions or more osteomas, it is necessary to evaluate for Gardner syndrome, a disorder consisting of osteoma, intestinal polyposis, multiple epidermoid cysts and dental anomalies [6]. The exact etiology of osteoma is still uncertain, with several theories explaining the process of osteoma formation through osteogenic neoplasm, infection and trauma [2,5]. In cranial osteoma, the best radiological modality for diagnosis is a CT scan which is superior to plain radiographs and MRI.

The lack of extracranial manifestation of osteoma knowledge often makes the diagnostic process more difficult, harboring numerous and possibly invasive diagnostic approaches, when in reality the nature of the disease is rather benign. In this paper, we report a case of osteoma at multiple sites including left ulna and bilateral fibula and detected by bone survey. Besides the rare location of osteomas, this report shows the importance of bone survey in evaluating patients with multiple osteomas. We reported our case in line with the SCARE 2020 guideline [16].

## 2 Case description

A 26-year-old female with bilateral pain in the jawbone and several areas of her head was referred to our hospital. The pain was dull, worse at night, and unaffected by activity. The patient denied any previous history of trauma, infection, or malignancy in her past medical history and in family history. The complaint dated back to when the patient was 5 years old, as the pain was first experienced and accompanied by a noticeable lump on her mandible which slowly grew in size but unfortunately was not sought for medical treatment.

**Fig. 1:**
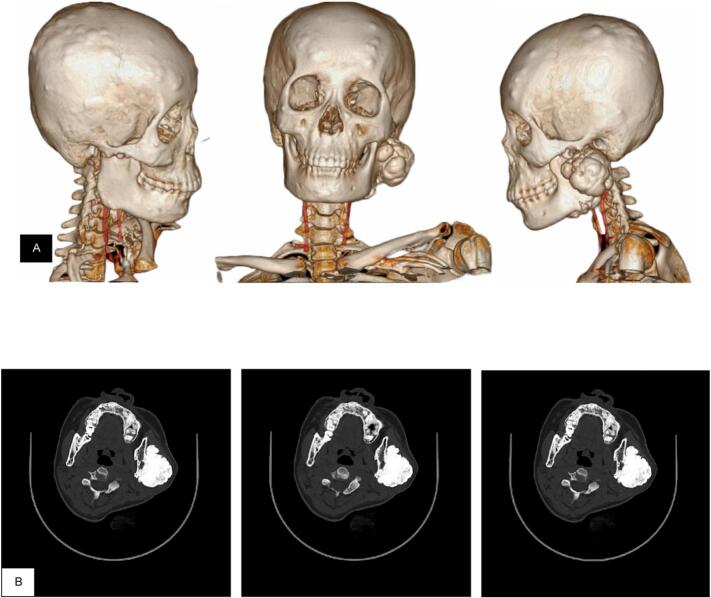
A. MSCT of pre-hemi-mandibulectomy: shows multiple rounded lesions, firm borders, and regular edges in bilateral parietal, temporal bilateral, and frontal bilateral. Multiple lesions of the mandibular region with 5.6 × 6.0 × 4.5 cm in size, B. CT cross-sectional image shows lesions in left mandibular region. The lesion is arising on the surface of the bone and homogenously dense.

As the primary complaint was manifested within the cranium, the referral was directed for the Oncology Surgery Department at our hospital. From physical examination, it was noted that several masses in the skull were present but the clearly noticeable mass was on the left mandible. The patient complained of pain but with a normal range of motion of the temporomandibular joint. Multi-slice Computed Tomography (MSCT) of the head was ordered which revealed bilateral parietal, temporal and frontal bone osteoma with ivory osteoma on both sides of mandible and right ethmoidal bone (Fig. 1). Specifically, the osteoma mass on her left mandible was massive, measuring at 5,6 × 6.0 × 4,5 cm.

**Fig. 2:**
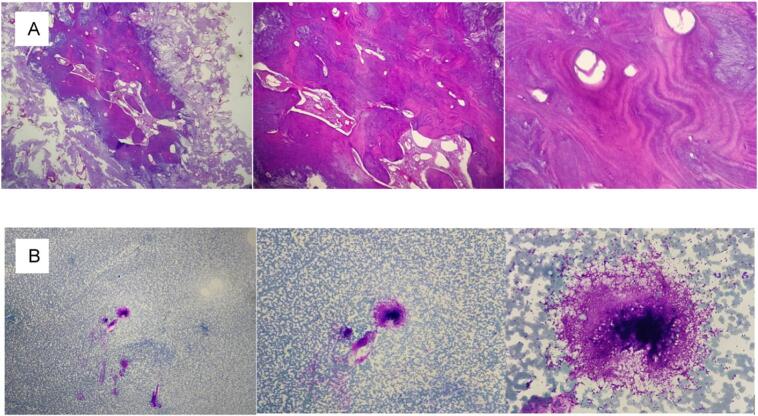
Histopathology of left mandible. A showed bone tissue consisting of compact and lamellar bone and absence of malignant cells. B the cytology results support the conclusion by only finding inflammatory cell and a cluster of fibroblasts. The conclusion is osteoma.

The oncology surgeon performed left hemi-mandibulectomy and sent the sample to pathology for further examination. As for the right mandible, ultrasound-guided fine needle aspiration was performed 2 months post-operative. Both pathology samples showed bone tissue consisting of compact and lamellar bone with various Haversian canals, and a distribution of inflammatory cells consisting of neutrophils, lymphocytes, and macrophages were found (Fig. 2). The final conclusion of osteoma was confirmed with no cellular characteristic for malignancy.

**Fig. 3:**
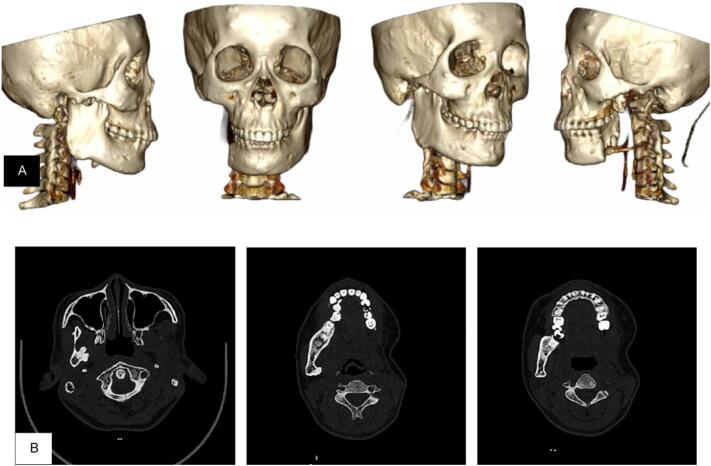
A & B 3D and cross-sectional MSCT shows no residual lesion in mandible sinistra post hemi mandibulectomy.

Fourteen months following the hemi-mandibulectomy, the patient complained of persisting pain on her right angle of mandibula, which a head MSCT was repeated. The latest MSCT showed no meaningful growth of lesions compared to the prior imaging (Fig. 3). The patient was then referred to the Orthopaedic and Traumatology Department for a more thorough imaging of possible skeletal lesion dissemination.

**Fig. 4:**
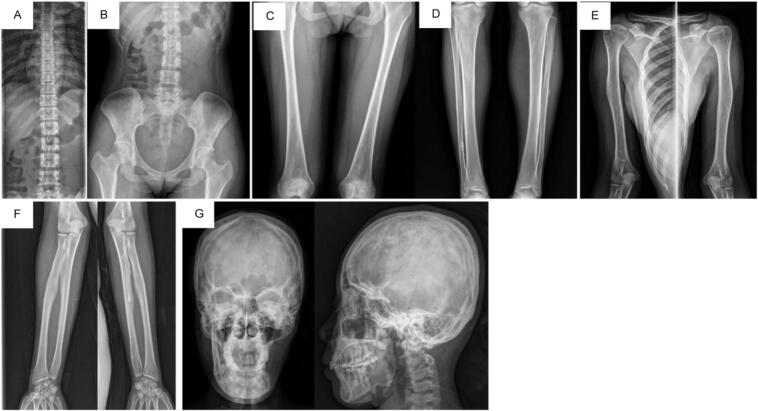
Bone surveys show lytic lesions of round, regular edges, in the cortex with narrow transition zones in the bones of the left ulna, right distal fibula and middle left fibula and suspected as osteoma.

A whole-body bone survey was performed and two extracranial lesions were visualized. Both were lytic lesions with regular margin and narrow transitional zone, each found on, left ulna, distal third of right fibula and medial third of left fibula (Fig. 4).

**Fig. 5:**
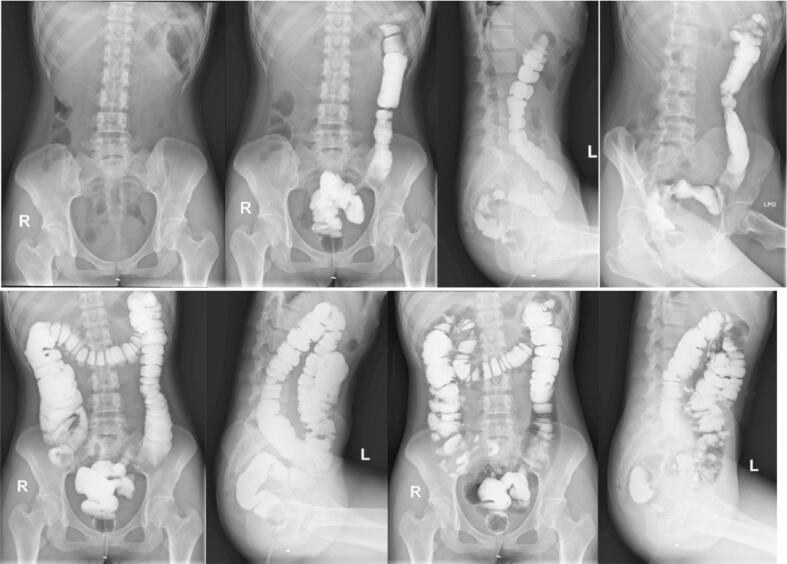
Colon in loop shows no polyps or intraluminal masses that exclude Gardner syndrome.

As multiple osteomas were found in various areas, we ordered for colon-in-loop examination and found no visible polyps or intraluminal masses in the colon were visualized (Fig. 5). This excludes the suspicion of Gardner's Syndrome. Combined with the patient's history and result of supporting examination we conclude the final diagnosis of the patients as osteoma with cranial and extracranial manifestation. We opted for monthly observation, which up to six months did not show progression of symptoms of the lower limbs.

## 3 Discussion

Osteoma is a benign, slowly developing tumor that can develop from either compact or cancellous bone [8,9]. The prevalence of osteomas is thought to be higher due to the underreporting given that the majority of osteomas are slow-growing and asymptomatic, accounting for 11 % of benign bone tumors and 1 % of all bone tumors, respectively [10]. The prevalence is similar between genders with higher occurrence in the second and third decades of life [11,12].

Although the exact etiology of osteoma is still unknown, some factors have been suggested to be responsible, including developmental anomalies of periosteum, genetic defects, reactive condition due to trauma or infection, and alteration of the calcium metabolism [3,13]. Our patient was unable to recall any trauma or infection preceding the development of her mandibular mass. Presentation of multiple craniofacial osteomas is often associated with Gardner's syndrome, a subtype of familial adenomatous polyposis caused by mutations in the APC gene. This autosomal dominant disorder typically manifests as multiple osteomas of the skull with systemic manifestations, such as intestinal polyposis, dental anomalies, and epidermoid cyst [6]. We screened the patient with colon in loop which showed no detectable intraluminal masses or polyps in the colon and Gardner's syndrome diagnosis was ruled out.

Osteoma can be differentiated by their manifesting site; peripheral osteomas on periosteum, central osteomas on the endosteum within the bone, and extra-skeletal osteomas on soft tissues, usually involving muscles [14]. Peripheral osteoma comprises the majority of osteoma cases, which attach to the cortical bone in a sessile or pedunculated manner. Osteoma predominantly affects bones formed by membranous ossification. The corpus, condyle, and mandibular angle are the most often affected areas by peripheral osteoma of the mandible [12]. Meanwhile, osteoma of the long bones is incredibly uncommon [2]. Interestingly, our patient presented with uncommon manifestation of both cranial and extracranial osteomas.

Craniofacial osteoma commonly manifested as swelling, although it may become noticeable to patients once facial disfigurement occurs [9]. Aside from aesthetic issues, osteoma typically produces no symptoms nor cause any discomfort to patients [5,9]. Hence, the diagnosis of osteoma may be challenging due to its asymptomatic nature. In some cases, the growing lesion gradually exert pressures to the surrounding structures and causes symptoms related to mass effect, as reported in this case. Patients with osteoma may present with local pain and headache due to impingement of adjacent neurovascular structures [5,9]. Predilection of osteoma in the mandible also disrupted the function of the temporomandibular joint (TMJ) and caused trismus [13].

The clinical finding is further confirmed with radiologic exam, which typically reveal a well-circumscribed radio-opaque mass, attached to diaphysis or meta-diaphysis of a long bone in a mushroom-like manner. Even though the diagnosis of a peripheral osteoma can typically be made with a conventional radiograph, computed tomography (CT) with three-dimensional reconstruction is the best imaging modality to localize the mass and plan for comprehensive management before surgical removal [1]. CT scan of an osteoma mass will show as a well-defined, frequently lobulated, homogenously hyperdense mass without soft tissue involvement [2]. With 3D reconstruction, CT scan may provide better clarity and more accurate localization. Additionally, CT images can be used to rule out Gardner's syndrome, which may be characterized by the presence of numerous osteomas, impacted extra teeth, and odontoma. We strongly suspected that our patient had the non-syndromic variety of multiple osteoma based on her CT scan result which did not reveal any of the other characteristics [3]. Meanwhile, MRI is able to visualize the hypointense lesion on all sequences, which follows the signal intensity of normal cortical bone without any indications of intramedullary extension or associated soft tissue components [2].

Due to absence of pain, some cases of osteoma are found incidentally from radiograph examination, which might explain the underreporting of the disease [5]. To anticipate the possibility of missed lesions, we ordered a whole-body bone survey for the patient. The bone survey visualized extracranial lesions on the left ulna, right fibula and medial third of the left fibula, even though patients had no complaints in the lower extremities. We believe this result highlight the benefit of routine bone survey as part of multiple osteoma patient care to reveal asymptomatic lesions that otherwise might go undetected.

Histopathology examinations of osteoma composed of compact/ivory type, cancellous, trabecular/spongy type, and mixed type similar to that of mature bone [3]. The marrow cavities in cancellous regions are usually well-vascularized, moderately cellular, and fibrous stroma. Osteoblasts, osteocytes, and normal haversian canals are commonly found here. Reflecting back on our patient, the tissue sample consisted of compact and lamellar bone with some inflammatory cells and showed no characteristics for malignancy, which are appropriate for the diagnosis of osteoma. Therefore, while our presented case demonstrated many characteristic features of osteoma, this case is very unusual in that (1) bone survey revealed fibular and ulnar manifestation, (2) the presence of both cranial and extracranial features, and (3) multiple osteomas suggestive of Gardner's syndrome but without other systemic manifestations.

Asymptomatic osteoma is usually treated conservatively with periodic follow-up and serial radiographs [13]. Surgical treatment is justified when lesion shows rapid growth suggestive of malignancy or produces significant loss of function in the affected areas [5]. Most common choice of treatment is surgical excision, which requires removal of the underlying cortex [15]. Despite having higher risk of fracture, surgery is linked to lower rate of recurrence. Implementing this rationale in mind, hemi-mandibulectomy was opted by the surgeon in this case. Along with surgical technique development, recent studies reported percutaneous CT-guided techniques, such as radio-frequency ablation which have comparable success rate to that of open surgery. Extra caution is required with this technique if the lesion is located near neurovascular bundles as they may complicate to osteonecrosis [16].

## 4 Conclusion

Osteoma is a benign tumor that is variable in presentation and may exist as an isolated lesion or part of Gardner syndrome. Although there are several known predilected sites, osteoma is rarely found in the fibula. The difficulty in diagnosis rooted from inconsistent pattern of presentation and symptoms which may not disturb patients to want to seek medical treatment. Evaluation of patients with multiple osteomas should be done thoroughly and we propose that bone surveys are valuable in ensuring potential involvement of other sites. It is also important to obtain histological nature of the lesion as therapy for osteoma differ and also based on the symptoms felt by the patient. Surgery might be the best option in symptomatic patients while periodic observation in asymptomatic patients is acceptable without significant lesion progression.

## Ethical approval

Ethical approval for this study was provided by The Medical and Health Research Ethics Committee (MHREC) Faculty of Medicine, Public Health and Nursing Universitas Gadjah Mada- Dr. Sarjito General Hospital on 20 July 2023 and the protocol number is KE/FK/1202/EC/2023

## Funding

This research received no external funding

## CRediT authorship contribution statement

Conceptualization (Y.A.P.P., R.M. K.K.S) Methodology (Y.A.P.P., R.M., K.K.S) Investigation (Y.A.P.P., R.M., K.K.S) Supervision (Y.A.P.P., R.M., K.W.T.H., E.K.D., A.W.P) Validation (Y.A.P.P., R.M., K.W.T.H., E.K.D., A.W.P) Writing original draft (Y.A.P.P., R.M., K.K.S).

## Guarantor

Y.A.P.P.

## Consent for publications

Written informed consent was obtained from the patient for publication of this case report and any accompanying images. A copy of the written consent is available for review by the Editor-in-Chief of this journal.

## Declaration of competing interest

The author declares that there is no conflict of interest. This case report did not receive any specific funding from agencies in the public, commercial, or for-profit sectors.

## Data Availability

Supporting data will be available upon reasonable request
